# Development of a Novel Algorithm to Identify People with High Likelihood of Adult Growth Hormone Deficiency in a US Healthcare Claims Database

**DOI:** 10.1155/2022/7853786

**Published:** 2022-06-18

**Authors:** Kevin C. J. Yuen, Anna Camilla Birkegard, Lewis S. Blevins, David R. Clemmons, Andrew R. Hoffman, Nicky Kelepouris, Janice M. Kerr, Jens M. Tarp, Maria Fleseriu

**Affiliations:** ^1^Barrow Pituitary Center, Barrow Neurological Institute and St. Joseph's Hospital and Medical Center, University of Arizona College of Medicine and Creighton School of Medicine, Phoenix, AZ, USA; ^2^Novo Nordisk A/S, Søborg, Denmark; ^3^Department of Neurosurgery, University of California, San Francisco, CA, USA; ^4^Department of Medicine, University of North Carolina School of Medicine, Chapel Hill, NC, USA; ^5^Department of Medicine, Stanford University, Stanford, CA, USA; ^6^Novo Nordisk Inc., Plainsboro, NJ, USA; ^7^Department of Endocrinology, University of Colorado Health Sciences Center, University of Colorado Denver, Anschutz Medical Campus, Aurora, CO, USA; ^8^Pituitary Center, Departments of Medicine and Neurological Surgery, Oregon Health and Science University, Portland, OR, USA

## Abstract

**Objective:**

Adult growth hormone deficiency (AGHD) is an underdiagnosed disease associated with increased morbidity and mortality. Identifying people who may benefit from growth hormone (GH) therapy can be challenging, as many AGHD symptoms resemble those of aging. We developed an algorithm to potentially help providers stratify people by their likelihood of having AGHD.

**Design:**

The algorithm was developed with, and applied to, data in the anonymized Truven Health MarketScan® claims database. *Patients*. A total of 135 million adults in the US aged ≥18 years with ≥6 months of data in the Truven database. *Measurements*. Proportion of people with high, moderate, or low likelihood of having AGHD, and differences in demographic and clinical characteristics among these groups.

**Results:**

Overall, 0.5%, 6.0%, and 93.6% of people were categorized into groups with high, moderate, or low likelihood of having AGHD, respectively. The proportions of females were 59.3%, 71.6%, and 50.4%, respectively. People in the high- and moderate-likelihood groups tended to be older than those in the low-likelihood group, with 58.3%, 49.0%, and 37.6% aged >50 years, respectively. Only 2.2% of people in the high-likelihood group received GH therapy as adults. The high-likelihood group had a higher incidence of comorbidities than the low-likelihood group, notably malignant neoplastic disease (standardized difference −0.42), malignant breast tumor (−0.27), hyperlipidemia (−0.26), hypertensive disorder (−0.25), osteoarthritis (−0.23), and heart disease (−0.22).

**Conclusions:**

This algorithm may represent a cost-effective approach to improve AGHD detection rates by identifying appropriate patients for further diagnostic testing and potential GH replacement treatment.

## 1. Introduction

Adult growth hormone deficiency (AGHD) is a rare endocrine disorder characterized by an abnormal decrease in growth hormone (GH) secretion [1]. Growth hormone deficiency (GHD) can develop and persist from childhood into adulthood, or be newly acquired in adulthood [2]. There are multiple potential causes of AGHD, including pituitary adenomas, craniopharyngiomas and their treatment with surgery and/or cranial radiotherapy, traumatic brain injury, genetic defects, congenital malformations, subarachnoid hemorrhage, dysgerminomas and other parasellar tumors, and infiltrative, inflammatory, and vascular diseases [1, 2].

AGHD is associated with alterations to body composition, including reduced lean body mass and increased abdominal adiposity, reduced muscle strength, decreased aerobic exercise capacity, and adverse changes to lipid and carbohydrate metabolism [3]. Patients with AGHD also frequently report impaired psychological well-being and potentially significant neuropsychiatric manifestations, such as impaired mood and concentration, and memory loss [4]. Furthermore, it is likely that AGHD contributes to increased cardiovascular (CV) morbidity and mortality compared with the general population, especially in patients with panhypopituitarism [5, 6].

Historically, it has been difficult to accurately estimate AGHD prevalence. It has been suggested that a reasonable estimate can be derived by combining prevalence data on pituitary macroadenoma (approximately 1 per 10,000 population) with cases of childhood-onset GHD persisting into adulthood [7]. This gives an overall estimated AGHD prevalence of 2–3 per 10,000 population [7]. Similarly, AGHD incidence is not well documented, but has been estimated at approximately 2 per 100,000 population, when cases with childhood-onset GHD persisting into adulthood are included [1, 8]. Such cases account for ∼15–20% of AGHD cases [1, 9]. These data probably underestimate the true incidence of AGHD, owing to factors including study-design limitations; the populations sampled, with an underrepresentation of older people and those with mild or not recognizable symptoms; insufficient information available in medical records; lack of referrals and diagnostic tests; and suboptimal clinical awareness. [8, 10, 11].

In appropriately selected patients, recombinant human GH therapy has been shown to be effective in treating AGHD by improving body composition, muscle strength, lipid profile, and quality of life [1]. The safety profile of GH treatment in AGHD is well established, with the final decision to treat AGHD requiring a careful clinical evaluation of the risks and benefits to the person [2]. Although consensus clinical guidelines recommend confirming a diagnosis of AGHD with a GH stimulation test, in an appropriate clinical context, before considering GH replacement therapy, [12] AGHD can also be accurately predicted through the presentation of multiple pituitary hormone deficiencies (≥3) in the presence of low serum age- and sex-adjusted insulin-like growth factor-I (IGF-I) [13]. However, identifying adults likely to have AGHD for diagnostic referral can be challenging [14]. Unlike in children, in whom GHD is relatively straightforward to identify because of decreased growth velocity and short stature, many of AGHD's clinical features are nonspecific and resemble aspects of normal aging. Moreover, diagnosis in adults is further challenged by the absence of a single, reliable biological marker [14]. The limitations in detection and the reported heterogeneity in AGHD detection rates worldwide suggest that AGHD is underdiagnosed [15].

This study reports the development of a novel algorithm using healthcare claims data to stratify people according to their likelihood of having AGHD and, thus, might help detect people with AGHD who may benefit from further diagnostic evaluation. Additionally, we describe the application of the algorithm to a US cohort and characterize the available demographic, clinical, and administrative data for the resulting likelihood groups.

## 2. Materials and Methods

### 2.1. Algorithm Development

The initial design of the algorithm was based on a combination of parameters including diagnosis codes, diagnostic tests, and medications (identified in AGHD guidelines [1, 2, 16, 17]). These parameters were adjusted by a set of logical rules, aiming to categorize the study cohort by their likelihood of having AGHD into three groups: high, moderate, and low. The study cohort comprised people included in the Truven Health MarketScan® Commercial Claims and Encounters Database and the Medicare Supplemental and Coordination of Benefits Database (hereafter referred to as the Truven database). The Truven database is de-identified and organized in a manner that ensures data privacy and only contains fully paid health claims. Information provided in the claims includes physician, hospital, and pharmacy claims with diagnoses, medications, and healthcare resource use.

The algorithm recognized diagnosis codes that are included in the International Classification of Diseases, Ninth Revision, Clinical Modification (ICD09CM) or International Classification of Diseases, Tenth Revision, Clinical Modification (ICD10CM). Furthermore, the algorithm used current procedural terminology (CPT) codes for diagnostic tests and anatomical therapeutic chemical (ATC) codes for medications. The logical rules included age and number of diagnoses, diagnostic tests, and prescribed medications.

The algorithm and related diagnosis code lists (included in the Supplementary Materials (Available here)) were refined further through an iterative stepwise process based on feedback from an expert committee, which included internal (employed by the study sponsor) and external (not employed or paid for by the study sponsor) endocrinology/pituitary specialists. During this stage of iterative refinement, the algorithm was applied to a random training cohort. The training cohort consisted of 10 million adults in the US aged ≥18 years as of 31 December 2017, with ≥6 months of data in the Truven database from the start date of 1 January 2001. The committee members reviewed the numbers yielded from the training set, adjusted the numbers based on subject matter expertise, and applied logical rules to optimize the algorithm and skip redundant steps. The algorithm was considered final once no further refinements were required by the expert committee.

### 2.2. Application of the Algorithm to a US Cohort

The final version of the algorithm was applied to the full study cohort of adults aged ≥18 years as of 31 December 2017 (the index date) with ≥6 months of data in the Truven database in a retrospective, cross-sectional study. The proportion of people classified with a high, moderate, or low likelihood of having AGHD by the study algorithm was defined as the number of living people in each respective likelihood group at the index date divided by the number in the overall study cohort. Demographic, clinical, and administrative data were described for the overall study cohort and for the three likelihood groups, capturing the incidence of comorbid conditions over the last year of observation. Effect size was calculated for the standardized difference between groups in the frequency of comorbidities. Given the binary nature of the comorbidity variables, a standardized difference of ≤0.1 was considered negligible [18].

## 3. Results

The algorithm development and results of its application are summarized in an infographic (Supplementary Materials (available here)).

### 3.1. Algorithm Development

The first iteration of the algorithm structure (without data) was presented to the expert committee. The algorithm structure (iteration 2) and results from its application to the training cohort were reviewed by the expert committee, and the algorithm and related diagnosis code lists were amended based on the committee's advice. The algorithm structure (iteration 3) and results from its application to the training cohort were reviewed by the expert committee, which recommended further changes to the algorithm and related diagnosis code lists. Following a review of the fourth iteration of the algorithm structure and results from its application to the training cohort, the expert committee recommended the addition of a second exclusion list of diagnoses to the algorithm. The revised algorithm structure (iteration 5) and results from its application to the training cohort were reviewed by the expert committee, which recommended an increase in the minimum number of pituitary hormone deficiencies (other than GH) in people aged ≥18 years from two to three, to increase the accuracy of the algorithm. The revised algorithm following this change (iteration 6) was considered final.

### 3.2. Final Algorithm Structure

**Figure 1:**
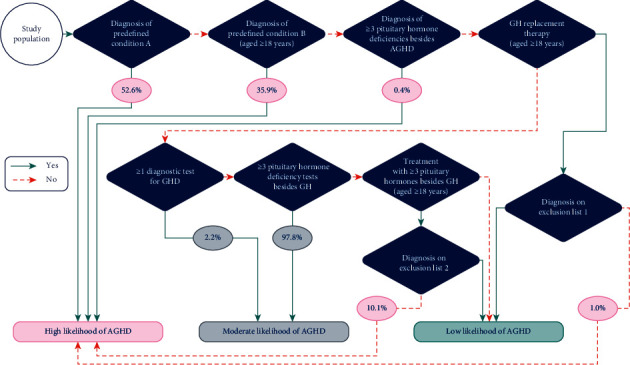
Schematic of a novel algorithm to categorize people by their likelihood of having AGHD using administrative claims data. The algorithm considered diagnosis codes, diagnostic tests, and treatments for each person in the study population. The flowchart consists of a number of guiding rules (diamonds), which result in the placement of each person into one of three groups by their likelihood of having AGHD. Each diamond is dependent on a set of codes (ICD10CM/ICD09CM for diagnoses, CPT for tests, and ATC for treatments), which, combined with logical rules dependent on age, number of prescriptions, or diagnoses, were used to determine whether each person obeys the rule, as indicated by a green (yes) or dashed orange (no) arrow. AGHD, adult growth hormone deficiency; ATC, anatomical therapeutic chemical; CPT, current procedural terminology; GH, growth hormone; GHD, growth hormone deficiency; ICD09CM, International Classification of Diseases, ninth revision, Clinical Modification; ICD10CM, International Classification of Diseases, tenth revision, Clinical Modification.

A schematic of the final algorithm structure is shown in Figure 1. For inclusion in the high-likelihood group, the final algorithm required at least one of the following: ≥1 diagnosis of predefined conditions (Table S1); diagnosis of ≥3 pituitary hormone deficiencies besides AGHD (Table S2); ≥1 prescription(s) for GH replacement therapy (aged ≥18 years, Table S3) and absence of a diagnosis on a predefined exclusion list (Tables S4 and S5); or treatment with ≥3 pituitary (or target gland) hormone replacements besides GH, such as sex hormones, corticosteroids for systemic use or thyroid preparations, as indicated by ≥1 prescription(s) for each hormone replacement within the same year (aged ≥18 years; Table S6), and absence of a diagnosis on a predefined exclusion list (Tables S4 and S5). The predefined conditions (Table S1) could have been diagnosed at any age (group *A*), or only after the age of 18 years (group *B*) to categorize a patient into the high-likelihood group.

People who did not satisfy criteria for the high-likelihood group but had either ≥1 diagnostic test for GHD (Table S7) or ≥3 pituitary hormone deficiency tests besides GH (Table S8), each with unknown results due to the nature of the database, were categorized in the moderate-likelihood group. Finally, people who did not satisfy any of the aforementioned criteria were categorized into the low-likelihood group.

### 3.3. Application of the Algorithm to a US Cohort

The overall study cohort in the Truven database consisted of 135 million people with ≥6 months of data between 1 January 2001 and 31 December 2017 (a total period of data coverage was 17 years). The mean (SD) observation period was 3.35 (3.04) years for all patients (5.20 [3.81], 5.70 [4.00], and 3.18 [2.90] years for the high-, moderate-, and low-likelihood groups, respectively). Overall, 0.5%, 6.0%, and 93.6% of those screened were found to have a high, moderate, or low likelihood of having AGHD, respectively. In the high-likelihood group, the majority (52.6%) were categorized based on diagnoses of predefined conditions from group *A*, followed by people classified based on conditions from group *B* (35.9%). In the moderate-likelihood group, most people (97.8%) were categorized on the basis of having ≥3 pituitary hormone deficiency tests besides GH (Figure 1).

The age and sex distribution of the study cohort is shown in Table 1. Overall, there was an even distribution of females and males (51.7% and 48.3%, respectively). The proportion of females in the high-, moderate-, and low-likelihood groups was 59.3%, 71.6%, and 50.4%, respectively. The high- and moderate-likelihood groups tended to be older than the low-likelihood group, with 58.3%, 49.0%, and 37.6% aged >50 years, respectively. The proportion of people in the high- and moderate-likelihood groups peaked in the age ranges of 60–70 and 50–60 years, respectively, before decreasing in the older age ranges. In contrast, the proportion of people with a low likelihood of AGHD was negatively correlated with age (Table 1).

The most common comorbidities among people in the high-likelihood group were hyperlipidemia (32.6%), hypertension (31.4%), and acute respiratory disease (30.2%) (Table 2). With the exception of some categories of neoplasms, there were similar patterns of comorbidities in the high- and moderate-likelihood groups (standardized difference [SD] <0.2). However, overall incidences of malignant neoplastic disease (23.9% vs 5.7%) and malignant tumor of the breast (8.8% vs 1.3%) were higher in the high- vs moderate-likelihood group (SD −0.34 and −0.24, respectively). Additionally, patients with central diabetes insipidus (CDI) were only present in the high-likelihood group (1.1%) and absent from the moderate- and low-likelihood groups. In comparison with the low-likelihood group, comorbidity rates were higher in the high- and moderate-likelihood groups across a broad range of illnesses.

Effect sizes indicated larger differences in comorbidities between the high- and low-likelihood groups than between the moderate- and low-likelihood groups, as anticipated. Notable differences between the high- and low-likelihood groups, in descending effect size, included malignant neoplastic disease (23.9% vs 2.5%; SD −0.42), malignant tumor of the breast (8.8% vs 0.5%; SD −0.27), hyperlipidemia (32.6% vs 14.6%; SD −0.26), hypertensive disorder (31.4% vs 14.6%; SD −0.25), osteoarthritis (17.1% vs 6.2%; SD −0.23), heart disease (15.6% vs 5.4%; SD −0.22), visual system disorder (23.9% vs 11.0%;SD −0.22), diabetes mellitus or impaired glucose tolerance (14.1% vs 5.7%; SD −0.18), depressive disorder (13.9% vs 5.6%; SD −0.19), and hematologic neoplasm (4.5% vs 0.3%; SD −0.19).

The most commonly applied diagnostic tests for GHD in the high-likelihood group, although infrequent, were IGF-I (somatomedin C; 19.2%) and human GH (somatotropin; 7.2%) serum levels; GH stimulation tests (including insulin tolerance) were rarely used (Table 3). The pattern of testing was similar among people in the moderate-likelihood group, but testing was performed less frequently. With regard to treatment, only 2.2% of patients in the high-likelihood group received GH replacement therapy as adults. Patients who received GH treatment as adults could not be categorized into the moderate-likelihood group owing to the algorithm structure (Figure 1).

## 4. Discussion

We have described the development of a novel algorithm to categorize people by their likelihood of having AGHD based on healthcare claims data. Application of the final algorithm to a large US cohort (135 million adults) yielded 0.5%, 6.0%, and 93.5% of the study cohort with a high, moderate, or low likelihood of having AGHD, respectively.

The proportion of people with a high likelihood of having AGHD observed in our study (0.5%) appears to be one order of magnitude larger than the estimated prevalence of AGHD of 2–3 per 10,000 in the literature. However, there are a number of potential factors that may explain this discrepancy. Firstly, the categorization of people into the high-likelihood group, based on healthcare claims data alone, is likely to have overestimated AGHD prevalence in our study, as not all conditions in the predefined group A cause complete GHD (i.e., unilateral cleft lip/palate), and most patients did not undergo confirmatory GHD testing [12]. Secondly, AGHD is underdiagnosed and under-reported, [1, 7–9] thereby leading to underestimated AGHD incidence and prevalence rates in the literature. This is further exacerbated by the lack of an AGHD-specific diagnostic code in the ICD10 lexicon and large databases. Lastly, some GHD-related tests are not routinely available or are not possible to perform in some clinical settings. Thus, the standard diagnostic criteria for establishing GHD are often not met in routine practice. The low proportion of patients in the high-likelihood group who received GH replacement therapy as adults (only 2.2%) suggests that the extent of AGHD under-treatment could be much higher than that reported by physicians in real-world clinical practice. [19].

While database studies have been used to study the epidemiology of chronic conditions, such as acromegaly, [20] to our knowledge, this is the first study of this kind in AGHD. The study algorithm was developed based on clinical guidelines and expert input using information available in healthcare claims databases as an additional tool to identify people with a high likelihood of having AGHD. In this report, we applied the algorithm to the anonymized Truven database; however, the algorithm could also be applied to nonanonymized databases, such as Medicare Claims Data, Veterans Affairs (VA), Veterans Health Administration Access Data, or the Kaiser Permanente healthcare system, to identify people who might benefit from further AGHD testing and GH treatment. However, the algorithm requires further validation, for example, by applying it on datasets from countries where GH is prescribed more commonly. If validated, the results of the algorithm could be used to identify criteria that determine a patient's likelihood of having AGHD. This information could ultimately be fed back to healthcare providers and aid in the future screening of appropriate patients for AGHD diagnostic testing.

While the sex ratio in the entire Truven cohort was balanced, there was a preponderance of females in the high- and moderate-likelihood groups. This result may be partially explained by the notion that females seek medical evaluation more readily than males. Additionally, perimenopausal and postmenopausal females were more likely to be categorized into high- and moderate-likelihood groups based on comorbidities and/or hormone replacement therapy.

People categorized with a high likelihood of having AGHD tended to be older with more comorbid conditions than those with a low likelihood. This observation was made despite the fact that the overall age distribution was skewed towards the 18–30-year age bracket (24.1% of the cohort), probably because the algorithm was based on many conditions with a higher prevalence in older adults. As both the high- and moderate-likelihood groups tended to be older than the low-likelihood group, this may have confounded an assessment of comorbidities. Accordingly, there were higher rates of comorbidities in the high- and moderate-likelihood groups than those in the low-likelihood group across nearly all disease categories, suggesting that the algorithm steps used to identify people with a high likelihood of AGHD may also identify markers of general illness and/or aging. This may, in part, be due to the steep decrease with patient age in the sensitivity of IGF-I serum levels as a diagnostic tool in AGHD [21]. However, the observed pattern of comorbidities in the high-likelihood group broadly reflects what would be expected in people with AGHD, adding to confidence in the accuracy of the algorithm. Between the high- and low-likelihood groups, the effect sizes revealed differences in comorbidities, the majority of which have been shown to be associated with AGHD in the literature or have a scientific rationale for such an association. Exceptions included obesity, metabolic syndrome, and osteoporosis, for which the observed differences were lower than expected. Small effect sizes between groups in continuous variables might suggest that the differences are not clinically relevant, or that the database may have limitations in terms of disease-control assessment, or that additional algorithm refinements are needed to accurately distinguish between the likelihood groups. However, the comorbidities investigated here were all binary variables, whereby small effect sizes can reflect large relative differences in comorbidity rates [18].

AGHD involves some well-defined adverse alterations to metabolism, body composition, bone mass, and joint physiology [7, 22]. Correspondingly, there were higher incidences of hyperlipidemia and osteoarthritis in the high- vs low-likelihood groups. Hypertension is estimated to be present in 25–30% of people with AGHD [23]. Hypothesized mechanisms contributing to the excess of hypertension seen in AGHD include endothelial dysfunction and vascular stiffening [23]. In this study, hypertensive disorder was present in 31.4% of the high-likelihood group compared with 14.6% of the low-likelihood group. The literature suggests that AGHD can enhance CV risk by increasing the prevalence of some well-known CV risk factors (central obesity, impaired lipid, and glucose profiles), in addition to some lesser-known CV surrogate risk markers such as pro-inflammatory cytokines, endothelial dysfunction, and oxidative stress [24]. In patients with hypopituitarism, CV risk may be further increased with chronic over- or under-replacement of glucocorticoids [1]. Consistent with the literature, we observed a higher incidence of heart disease in the high- vs low-likelihood group in this study.

GH also plays an important role in blood glucose regulation, with impaired glucose metabolism, insulin resistance, and fasting hyperinsulinemia reported in people with AGHD [24, 25]. The increase in abdominal obesity associated with AGHD is likely to contribute to the reduced insulin sensitivity observed in some people with AGHD [7]. Furthermore, the prevalence of diabetes mellitus has been demonstrated to be higher in people with AGHD than in the general population [26]. Consistent with these observations, the incidence of impaired glucose tolerance and diabetes mellitus was associated with increasing likelihood of having AGHD in our study. The elevated incidence of diabetes mellitus is also likely to have contributed to the increased incidence of visual system disorders observed in the high-likelihood group, as the diagnoses were largely associated with diabetic retinopathy and glaucoma.

The incidence of malignancies was increased with each higher likelihood category. While this observation could be partially influenced by the younger average age of the low-likelihood group, the differences between the high- and moderate-likelihood groups with similar age profiles are likely to be related to other factors. Our observation is consistent with previous reports of a significantly higher risk of cancer morbidity in patients with AGHD compared with controls [5]. The incidence of cancer was 2–5 times higher in adults with AGHD who had not received irradiation compared with non-AGHD controls [5]. The relationship between AGHD and the risk of cancer is unclear; however, it may involve obesity, [27] diabetes/insulin resistance [28], and/or low levels of IGF-I [29]. Obesity has been linked to an increased risk of cancer at 13 anatomical locations, including colon, thyroid, and postmenopausal breast cancer [30]. Similarly, type 2 diabetes has been linked to cancer at 20 different cancer sites, with the most robust evidence associating it with intrahepatic cholangiocarcinoma, breast, colorectal, and endometrial cancer [31]. The association between high levels of IGF-I and an increased risk of cancer has been well documented; however, there is a small amount of evidence to suggest that low IGF-I levels may also be associated with increased cancer mortality [29, 32].

The high- and moderate-likelihood groups had a higher incidence of acute respiratory disease than the low-likelihood group. There is a small amount of evidence to suggest a potential, but not necessarily causal, link between acute respiratory disease and AGHD [33–35]. However, a limitation of the database is that the types of diseases that fall under the category of “acute respiratory disease” are not specified. This makes it challenging to determine whether there is a link with AGHD or other patient characteristics within the likelihood groups. For example, increasing age and obesity (GHD-related conditions) have been widely reported as risk factors for acute respiratory distress syndrome [36, 37], while acute respiratory infections are more prevalent in younger adults [38].

Living with untreated AGHD can affect overall quality of life. Reported symptoms include depression, lack of energy, impaired concentration and memory, social isolation, body-image dissatisfaction, and anxiety. In this study, depressive disorder was more common in people categorized with high likelihood of AGHD. Data from clinical trials suggest that GH therapy leads to improvements in quality of life in patients with AGHD; however, the evidence is not yet conclusive [39].

The strengths of this study lie within the rigorous and robust development process for the algorithm that included the use of a training cohort, multiple iterations, and expert clinician feedback. In addition, the Truven database provided a large sample size.

Our study has several limitations. Firstly, the algorithm cannot provide a diagnosis of AGHD; instead, it identifies people with a high likelihood for having AGHD. It is not possible to validate the algorithm using the Truven data owing to the lack of access to primary data, such as medical records, and the anonymized nature of this database. Furthermore, there are limited longitudinal data for each person in the Truven database, owing to frequent switches of health insurance, resulting in potential gaps in medical history. It should also be noted that the data in the Truven database were collected from a range of different healthcare professionals, and thus, differences in the diagnostic work-up and care are to be expected. For example, the most commonly used diagnostic tools were serum levels of IGF-I and GH levels in fasted, rested patients, whereas the more reliable GH stimulation tests were rarely used. Furthermore, the measured serum IGF-I and GH levels were not available from the database. This is because data in the Truven database were collected for administrative purposes rather than for scientific use, and misclassification and registration errors may be present. Furthermore, the Truven study population may not be representative of the entire US population, as the database only includes working-age people covered by private insurance. It is likely that, owing to the healthy-worker effect (a selection bias created by studying actively employed people), our findings may also be biased in terms of the overall proportions, demographics, and comorbidities among the likelihood groups compared with the general population. Finally, the algorithm design did not include CDI or treatment with vasopressin. It has been reported that, in patients with CDI, at least one anterior pituitary axis is also commonly affected [40]. Interestingly, despite this limitation, all patients with CDI were successfully categorized into the high-likelihood group.

The presented algorithm represents a novel method to stratify people according to their likelihood of having AGHD. Given the presumed under-diagnosis of AGHD, such a tool could be useful for healthcare providers. Specifically, this approach could provide a cost-effective screening method to help identify people who could potentially benefit from further GHD testing and possible GH treatment. It may also open areas for the study of comorbidities associated with AGHD and facilitate comparative analyses of the different likelihood groups between databases. Lastly, a prospective study showing the proportion of confirmed AGHD diagnoses among the various likelihood groups could be useful for confirmation and further refinement of the algorithm.

In conclusion, we report the development of a novel algorithm to categorize people by their likelihood of having AGHD using healthcare claims data, which could be used to identify people who could potentially benefit from further AGHD diagnostic testing and GH treatment. This algorithm may represent a cost-effective approach to addressing the under-diagnosis of GHD in adults.

## Figures and Tables

**Table 1:** Age and sex distribution of the study cohort overall and by the likelihood of having AGHD.

	Likelihood of having AGHD (%)			Study cohort (%)
	High	Moderate	Low	
All	0.48	5.98	93.55	100.00
Female	0.28	4.28	47.15	51.71
Male	0.20	1.70	46.39	48.29
Age group (years)				
>18–≤30	0.06	0.63	23.43	24.12
>30–≤40	0.05	0.97	18.25	19.27
>40–≤50	0.08	1.45	16.72	18.25
>50–≤60	0.12	1.79	17.29	19.19
>60–≤70	0.13	1.03	13.99	15.14
>70–≤80	0.03	0.12	3.84	3.99
>80	0.00	0.00	0.03	0.03

Percentages in the likelihood groups were rounded to 2 decimal points. %, percentage of people from the study cohort; AGHD, adult growth hormone deficiency.

**Table 2:** Comorbidities of the study cohort by the likelihood of having AGHD.

	Likelihood of having AGHD (%)			Standardized difference		
	High	Moderate	Low	High vs low	High vs moderate	Moderate vs low
Cardiovascular						
Cerebrovascular disease	3.7	1.8	0.8	−0.14	−0.08	−0.06
Hypertensive disorder	31.4	25.9	14.6	−0.25	−0.07	−0.18
Heart disease	15.6	10.7	5.4	−0.22	−0.09	−0.13
Atrial fibrillation	1.9	1.2	0.6	−0.08	−0.04	−0.04
Coronary arteriosclerosis	4.6	3.1	1.8	−0.11	−0.05	−0.06
Heart failure	2.0	1.1	0.6	−0.09	−0.05	−0.04
Ischemic heart disease	2.7	1.8	1.0	−0.09	−0.04	−0.05
Peripheral vascular disease	1.5	1.1	2.2	0.04	−0.03	0.06
Pulmonary embolism or venous thrombosis	2.5	1.1	0.5	−0.12	−0.07	−0.05
Endocrine/metabolic/nutrition						
Anorexia nervosa or malnutrition	0.6	0.3	0.1	−0.07	−0.04	−0.03
Diabetes mellitus or impaired glucose tolerance	14.1	11.8	5.7	−0.20	−0.05	−0.15
Hyperlipidemia	32.6	28.8	14.6	−0.26	−0.05	−0.22
Metabolic syndrome X	1.3	1.4	0.3	−0.08	0.01	−0.09
Obesity	9.4	9.9	3.7	−0.16	0.01	−0.17
Musculoskeletal						
Osteoarthritis	17.1	14.9	6.2	−0.23	−0.04	−0.19
Osteoporosis	3.8	2.0	0.8	−0.14	−0.08	−0.07
Neoplasms						
Hematologic neoplasm	4.5	0.8	0.3	−0.19	−0.16	−0.05
Neoplasm of thyroid gland	1.4	0.7	0.2	−0.10	−0.05	−0.06
Neoplasm of bone	2.2	0.3	0.1	−0.13	−0.12	−0.02
Malignant neoplastic disease^a^	23.9	5.7	2.5	−0.42	−0.34	−0.11
Malignant lymphoma	1.6	0.3	0.1	−0.11	−0.09	−0.03
Malignant tumor of breast	8.8	1.3	0.5	−0.27	−0.24	−0.06
Malignant tumor of colon	1.2	0.2	0.1	−0.09	−0.09	−0.01
Malignant tumor of lung	2.1	0.2	0.1	−0.13	−0.13	−0.01
Primary malignant neoplasm of prostate	1.7	0.5	0.3	−0.10	−0.08	−0.03
Renal						
Renal impairment	3.5	2.0	0.9	−0.13	−0.06	−0.06
Respiratory						
Acute respiratory disease	30.2	29.6	20.1	−0.14	−0.01	−0.14
Chronic obstructive lung disease	3.6	1.8	1.0	−0.12	−0.08	−0.05
Pneumonia	3.4	1.7	1.0	−0.11	−0.08	−0.04
Other						
Dementia	0.5	0.3	0.1	−0.04	−0.02	−0.02
Depressive disorder	13.9	13.3	5.6	−0.19	−0.01	−0.18
Visual system disorder	23.9	19.0	11.0	−0.22	−0.08	−0.15

^a^Malignant neoplastic disease comprises malignant lymphoma; malignant neoplasms of anorectum, breast, colon, lung, or urinary bladder; or primary malignant neoplasm of prostate. %, percentage of people from the respective likelihood group; AGHD, adult growth hormone deficiency.

**Table 3:** Diagnostic tests for GHD in the study cohort by the likelihood of having AGHD.

	Likelihood of having AGHD (%)	
	High	Moderate
IGF-I serum level	19.2	4.3
GH serum level	7.2	1.0
GH stimulation test (arginine/levodopa)	0.3	0.0
GH stimulation test (glucagon)	0.1	0.1
GH stimulation test (insulin tolerance)	0.1	0.0

Data shown for the high- and moderate-likelihood groups. %, percentage of people from the respective likelihood group; AGHD, adult growth hormone deficiency; GH, growth hormone; GHD, growth hormone deficiency; IGF-I, insulin-like growth factor-I.

## Data Availability

Data subject to third-party restrictions. The data that support the findings of this study are available from a third party (International Business Machines Corporation). Restrictions apply to the availability of these data, which were used under license for this study. Data can be acquired through licenses from the vendor.

## Supplementary Materials

Supplementary Materials Table S1. Diagnoses belonging to groups *A* and *B* considered by the algorithm. Table S2. Diagnoses of pituitary hormone deficiencies other than AGHD considered by the algorithm. Table S3. Growth hormone treatments considered by the algorithm. Table S4. Diagnoses considered by the algorithm—exclusion list 1. Table S5. Diagnoses considered by the algorithm—exclusion list 2. Table S6. Treatment with pituitary hormones (other than growth hormone) considered by the algorithm. Table S7. Diagnostic tests for GHD considered by the algorithm. Table S8. Tests for pituitary hormone deficiencies other than growth hormone considered by the algorithm.
