# Supplementary material for: Development of a Novel Algorithm to Identify People with High Likelihood of Adult Growth Hormone Deficiency in a US Healthcare Claims Database
Source: Int J Endocrinol. 2022 Jun 18;2022:7853786. doi: 10.1155/2022/7853786 (PMC9233577; doi:10.1155/2022/7853786)
Supplement: Supplementary Materials — Table S1. Diagnoses belonging to groups A and B considered by the algorithm. Table S2. Diagnoses of pituitary hormone deficiencies other than AGHD considered by the algorithm. Table S3. Growth hormone treatments considered by the algorithm. Table S4. Diagnoses considered by the algorithm—exclusion list 1. Table S5. Diagnoses considered by the algorithm—exclusion list 2. Table S6. Treatment with pituitary hormones (other than growth hormone) considered by the algorithm. Table S7. Diagnostic tests for GHD considered by the algorithm. Table S8. Tests for pituitary hormone deficiencies other than growth hormone considered by the algorithm. [file 7853786.f1.zip › Supplementary Material 1_pdf.pdf]

Supplementary Material 1

Table S1. Diagnoses belonging to groups A and B considered by the algorithm.

Group	ICD version	Diagnosis name	Diagnosis code
A	ICD10CM	Multifocal and multisystemic (disseminated) Langerhans-cell histiocytosis	C96.0
A	ICD10CM	Hypopituitarism	E23.0
A	ICD10CM	Septo-optic dysplasia	Q04.4
A	ICD10CM	Cleft hard palate	Q35.1
A	ICD10CM	Cleft soft palate	Q35.3
A	ICD10CM	Cleft hard palate with cleft soft palate	Q35.5
A	ICD10CM	Cleft uvula	Q35.7
A	ICD10CM	Cleft palate, unspecified	Q35.9
A	ICD10CM	Cleft lip, bilateral	Q36.0
A	ICD10CM	Cleft lip, median	Q36.1
A	ICD10CM	Cleft lip, unilateral	Q36.9
A	ICD10CM	Cleft hard palate with bilateral cleft lip	Q37.0
A	ICD10CM	Cleft hard palate with unilateral cleft lip	Q37.1
A	ICD10CM	Cleft soft palate with bilateral cleft lip	Q37.2
A	ICD10CM	Cleft soft palate with unilateral cleft lip	Q37.3
A	ICD10CM	Cleft hard and soft palate with bilateral cleft lip	Q37.4
A	ICD10CM	Cleft hard and soft palate with unilateral cleft lip	Q37.5
A	ICD10CM	Unspecified cleft palate with bilateral cleft lip	Q37.8
A	ICD10CM	Unspecified cleft palate with unilateral cleft lip	Q37.9
A	ICD10CM	Malignant neoplasm of pineal gland	C75.3
A	ICD10CM	Benign neoplasm of pineal gland	D35.4
A	ICD10CM	Neoplasm of uncertain behavior of pineal gland	D44.5

A	ICD10CM	Neoplasm of uncertain behavior of craniopharyngeal duct	D44.4
A	ICD09CM	Neoplasm of uncertain behavior of pituitary gland and craniopharyngeal duct	237.0
A	ICD09CM	Letterer-Siwe disease, unspecified site, extranodal and solid organ sites	202.50
A	ICD09CM	Pituitary dwarfism	253.3
A	ICD09CM	Panhypopituitarism	253.2
A	ICD09CM	Other specified congenital anomalies of brain	742.4
A	ICD09CM	Cleft palate, unspecified	749.00
A	ICD09CM	Cleft palate, unilateral, incomplete	749.02
A	ICD09CM	Cleft lip, unspecified	749.10
A	ICD09CM	Cleft lip, unilateral, complete	749.11
A	ICD09CM	Cleft lip, unilateral, incomplete	749.12
A	ICD09CM	Cleft lip, bilateral, complete	749.13
A	ICD09CM	Cleft lip, bilateral, incomplete	749.14
A	ICD09CM	Cleft palate with cleft lip, unspecified	749.20
A	ICD09CM	Cleft palate with cleft lip, unilateral, complete	749.21
A	ICD09CM	Cleft palate with cleft lip, unilateral, incomplete	749.22
A	ICD09CM	Cleft palate with cleft lip, bilateral, complete	749.23
A	ICD09CM	Cleft palate with cleft lip, bilateral, incomplete	749.24
A	ICD09CM	Personal history of irradiation, presenting hazards to health	V15.3
A	ICD09CM	Malignant neoplasm of pineal gland	194.4
A	ICD09CM	Benign neoplasm of pineal gland	227.4
A	ICD09CM	Neoplasm of uncertain behavior of pineal gland	237.1
B	ICD10CM	Drug-induced hypopituitarism	E23.1
B	ICD10CM	Hypothalamic dysfunction, not elsewhere classified	E23.3
B	ICD10CM	Other disorders of pituitary gland	E23.6
B	ICD10CM	Disorder of pituitary gland, unspecified	E23.7

B	ICD10CM	Postprocedural hypopituitarism	E89.3
B	ICD10CM	Iatrogenic pituitary disorders	253.7
B	ICD09CM	Unspecified disorder of the pituitary gland and its hypothalamic control	253.9
B	ICD09CM	Other anterior pituitary disorders	253.4
B	ICD09CM	Other disorders of the pituitary and other syndromes of diencephalohypophyseal origin	253.8

ICD, International Classification of Diseases; ICD09CM, International Classification of Diseases, Ninth Revision, Clinical Modification; ICD10CM, International Classification of Diseases, Tenth Revision, Clinical Modification.

Table S2. Diagnoses of pituitary hormone deficiencies other than AGHD considered by the algorithm.

ICD version	Diagnosis name	Diagnosis code
ICD10CM	Diabetes insipidus	E23.2
ICD10CM	Secondary amenorrhea	N91.1
ICD10CM	Secondary oligomenorrhea	N91.4
ICD10CM	Benign neoplasm of pituitary gland	D35.2
ICD10CM	Galactorrhea not associated with childbirth	N64.3
ICD10CM	Unspecified adrenocortical insufficiency	E27.40
ICD09CM	Diabetes insipidus	253.5
ICD09CM	Benign neoplasm of pituitary gland	227.3
ICD09CM	Glucocorticoid deficiency	255.41

AGHD, adult growth hormone deficiency; ICD, International Classification of Diseases; ICD09CM, International Classification of Diseases, Ninth Revision, Clinical Modification; ICD10CM, International Classification of Diseases, Tenth Revision, Clinical Modification.

Table S3. Growth hormone treatments considered by the algorithm.

ATC code	ATC name
H01AC01	Somatropin
H01AC02	Somatrem

ATC, anatomical therapeutic chemical.

Table S4. Diagnoses considered by the algorithm – exclusion list 1.

ICD version	Diagnosis name	Diagnosis code
ICD10CM	Acromegaly and pituitary gigantism	E22.0
ICD10CM	Pituitary-dependent Cushing disease	E24.0
ICD10CM	Drug-induced Cushing syndrome	E24.2
ICD10CM	Ectopic ACTH syndrome	E24.3
ICD10CM	Alcohol-induced pseudo-Cushing syndrome	E24.4
ICD10CM	Other Cushing syndrome	E24.8
ICD10CM	Cushing syndrome, unspecified	E24.9
ICD09CM	Acromegaly and gigantism	253.0
ICD09CM	Cushing syndrome	255.0

ACTH, adrenocorticotropic hormone; ICD, International Classification of Diseases; ICD09CM, International Classification of Diseases, Ninth Revision, Clinical Modification; ICD10CM, International Classification of Diseases, Tenth Revision, Clinical Modification.

Table S5. Diagnoses considered by the algorithm – exclusion list 2.

ICD version	Diagnosis name	Diagnosis code(s)
ICD10CM	Dermatitis and eczema	L20–L30
ICD10CM	Lichen planus	L43
ICD10CM	Bullous pemphigoid	L12.0
ICD10CM	Inflammatory polyarthropathies	M05–M14
ICD10CM	Psoriasis	L40
ICD10CM	Noninfective enteritis and colitis	K50-52
ICD10CM	Primary adrenocortical insufficiency	E27.1
ICD10CM	Autoimmune hepatitis	K75.4
ICD10CM	Adrenogenital disorder, unspecified	E25.9
ICD10CM	Other hypothyroidism	E03
ICD10CM	Atrophy of vulva	N90.5
ICD09CM	Other inflammatory conditions of skin and subcutaneous tissue	690–698
ICD09CM	Lichen planus	697.0
ICD09CM	Pemphigoid	694.5
ICD09CM	Arthropathies and related disorders	710–719
ICD09CM	Other psoriasis	696.1
ICD09CM	Autoimmune hepatitis	571.42
ICD09CM	Adrenogenital disorders	255.2
ICD09CM	Congenital hypothyroidism	243
ICD09CM	Atrophy of vulva	624.1

ICD, International Classification of Diseases; ICD09CM, International Classification of Diseases, Ninth Revision, Clinical Modification; ICD10CM, International Classification of Diseases, Tenth Revision, Clinical Modification.

Table S6. Treatment with pituitary hormones (other than growth hormone) considered by the algorithm.

Group	ATC code	ATC name
Sex hormones	G03B	Androgens
Sex hormones	G03C	Estrogens
Sex hormones	G03D	Progesterone
ACTH	H02	Corticosteroids for systemic use
Thyroxine	H03A	Thyroid preparations

Only people with ≥2 prescriptions from ≥2 different groups within 1 year were considered by the algorithm.

ACTH, adrenocorticotropic hormone; ATC, anatomical therapeutic chemical.

Table S7. Diagnostic tests for GHD considered by the algorithm.

Test type	Test name	CPT code
Stimulation	Insulin tolerance test	80435
Stimulation	Glucagon stimulation test	82943
Stimulation	Arginine/levodopa stimulation test	80428
Laboratory	Growth hormone serum level	83003
Laboratory	IGF-I serum level	84305

CPT, current procedural terminology; GHD, growth hormone deficiency; IGF-I, insulin-like growth factor-I.

Table S8. Tests for pituitary hormones deficiencies other than growth hormone considered by the algorithm.

Group	Test name	Hormone measured	CPT code
Sex hormone	LH	LH	83002
Sex hormone	FSH	FSH	83001
Sex hormone	LH/FSH	Gonadotropin panel	80426
ACTH	ACTH	ACTH	82024
ACTH	ACTH with cortisol	ACTH stim panel	80400<br>82533
TSH	TSH	TSH	84443
Combined	Combined anterior pituitary panel	ACTH, TSH, cortisol, LH, FSH, prolactin, human growth hormone	80418
Sex hormone	Testosterone (done with LH/FSH) in men	Testosterone	80414<br>84403
Sex hormone	Estradiol (done with LH/FHS) in women	Estradiol	80415<br>82670
Sex hormone	Free testosterone	Testosterone	84402
Sex hormone	Bioactive testosterone	Testosterone	84410
TSH	Free T4	T4	84439
Sex hormone	Prolactin	Prolactin	84146

ACTH, adrenocorticotropic hormone; CPT, current procedural terminology; FSH, follicle-stimulating hormone; LH, luteinizing hormone; T4, thyroxine; TSH, thyroid-stimulating hormone.
