# Supplementary material for: Development of a Novel Algorithm to Identify People with High Likelihood of Adult Growth Hormone Deficiency in a US Healthcare Claims Database
Source: Int J Endocrinol. 2022 Jun 18;2022:7853786. doi: 10.1155/2022/7853786 (PMC9233577; doi:10.1155/2022/7853786)
Supplement: Supplementary Materials — Table S1. Diagnoses belonging to groups A and B considered by the algorithm. Table S2. Diagnoses of pituitary hormone deficiencies other than AGHD considered by the algorithm. Table S3. Growth hormone treatments considered by the algorithm. Table S4. Diagnoses considered by the algorithm—exclusion list 1. Table S5. Diagnoses considered by the algorithm—exclusion list 2. Table S6. Treatment with pituitary hormones (other than growth hormone) considered by the algorithm. Table S7. Diagnostic tests for GHD considered by the algorithm. Table S8. Tests for pituitary hormone deficiencies other than growth hormone considered by the algorithm. [file 7853786.f1.zip › Supplementary Material 2_infographic pdf.pdf]

What is AGHD?

A rare endocrine disorder, characterized by the deficiency of GH that can persist from childhood into adulthood or be newly acquired in adulthood[1,2]

- Clinical effects include abnormal body composition, impaired cognitive function, and reduced QoL[3,4]
- Identifying adults likely to have AGHD for diagnostic referral can be challenging, as there is no single biomarker and many clinical features resemble aspects of normal aging[5]
- Recombinant human GH therapy has been consistently demonstrated as beneficial for the treatment of AGHD, with low risks[1]
- There is limited epidemiological information on the prevalence within a large US population

Iterative algorithm development process

- Clinical guidelines[6–8]
- Draft algorithm
- Testing
- Expert clinical input
- Revision

Final algorithm to categorize people by their likelihood of having AGHD

Application of the final algorithm to an administrative healthcare database

135 million people from the US

	High likelihood of AGHD	Moderate likelihood of AGHD	Low likelihood of AGHD
Share	0.5%	6.0%	93.6%
Female	59.3%	71.6%	50.4%
Hyperlipidemia	32.6%	28.8%	27.8%
≥50 years old	58.3%	49.0%	37.6%
Hypertension	31.4%	25.9%	14.6%
GH therapy as adults	2.2%	0.0%	0.0%
Impaired glucose tolerance/diabetes*	14.1%	11.8%	5.7%

*diabetes mellitus

AGHD, adult growth hormone deficiency; GH, growth hormone; QoL, quality of life

Date of preparation: May 2021. Novo Nordisk Health Care AG, Zurich, Switzerland.

References 1. Yuen *et al. Endroc Pract* 2019;25:1191–232; 2. Molitch *et al. J Clin Endocrinol Metab* 2011;96:1587–609; 3. Melmed. *N Engl J Med* 2019;380:2551–62; 4. Gupta. *Indian J Endocrinol Metab* 2011;15 Suppl 3: S197–S202; 5. Reed *et al. Front Endocrinol* 2013;4:64; 6. Boguszewski. *Rev Argent Endocrinol Metab* 2010;47:30–8; 7. Giustina *et al. J Endocrinol Invest* 2008;31:820–38; 8. Ho. *Eur J Endocrinol* 2007;157:695–700.
